# Diagnostic concordance of emergency doctor-performed bedside ultrasonography versus specialist-performed echo-Doppler ultrasonography in the diagnosis of deep venous thrombosis of lower limbs

**DOI:** 10.1186/cc14214

**Published:** 2015-03-16

**Authors:** DL Ly-Pen, JP Penedo Alonso, MS Sánchez Perez, FR Roldán Moll, MZ Zamorano Serrano, LD Díaz Vidal, SJ Justo

**Affiliations:** 1 Southend University Hospital, Westcliff-on-Sea, UK; 2 Hospital Universitario Ramón y Cajal, Madrid, Spain; 3 Instituto Ramón y Cajal de Investigación Sanitaria (IRYCIS), Madrid, Spain

## Introduction

Deep venous thrombosis (DVT) is an increasing major cause of mortality and morbidity. There is a need for quick, easy, cheap, convenient and reliable diagnostic tools. The objectives were to ascertain the diagnostic concordance of emergency doctor-performed ultrasound (EDUS) of the lower extremities with specialist doctor-performed (radiologist or vascular surgeon) echo-Doppler (SDED), in the diagnosis of DVT, and to identify possible causes of nonconcordance.

## Methods

A prospective, multicentre study. Adult patients (>18 years old) with clinical suspicion of DVT, with high or moderate risk (on Wells scoring), or low risk with increased D-dimer levels, were eligible for the study. Emergency doctors performed two EDUS in femoral and popliteal areas (these results were blinded). After this, echo-Doppler was performed by specialist doctors. Both procedures were done within 24 hours of each other. The final result was considered nonconcordant if one or both of the EDUS did not match with the SDED. These SDED were used as reference (as standard clinical practice).

## Results

From September 2013 to September 2014, a total of 328 patients were enrolled. Fifty-one investigators from seven hospitals performed the EDUS. Each patient had the EDUS (femoral and popliteal areas) and SDED (also in femoral and popliteal areas). Of 328 pairs of US studies, 37 were nonconcordant between EDUS and SDED. Two EDUS were incomplete, so the concordance analysis was performed with 326 US studies, with 35 discordant. The percentage of agreement between EDUS and SDED was 89.26%. The kappa index was 0.76 (95% CI = 0.69 to 0.84), and this means a substantial agreement.

## Conclusion

There is substantial agreement between EDUS and SDED in the diagnosis of DVT, in routine clinical practice. This confirms the results of previous papers. The largest nondiagnostic concordance in thrombus occurs in the early performances of emergency doctors, especially until the fifth performance. After the sixth one, the incidence of mismatches falls dramatically. It seems advisable to mentor the training programs with at least five shadowed performances in order to lower the incidence of mistakes.

